# Synthesis and characterization of activated carbon from sugar beet residue for the adsorption of hexavalent chromium in aqueous solutions

**DOI:** 10.1039/d0ra09644j

**Published:** 2021-02-24

**Authors:** Jiaming Zhao, Lihua Yu, Feng Zhou, Huixia Ma, Kongyan Yang, Guang Wu

**Affiliations:** School of Chemistry and Materials Sciences, Research Institute of Crop Science, Heilongjiang University Harbin 150080 China wu.guang@163.com; Dalian Research Institute of Petroleum and Petrochemicals, SINOPEC Dalian 116045 China

## Abstract

A series of micro–mesoporous activated carbons (ACs) were prepared from sugar beet residue by a two-step method including KOH chemical activation and were used for Cr(vi) removal from aqueous solutions. Several characterization techniques, including SEM, TEM, N_2_ adsorption, XRD, FTIR, and Raman spectroscopy, were used to determine the chemical and physical characteristics of the ACs, and the adsorption properties of the ACs were tested. The results indicated that the high specific surface area of the ACs reached 2002.9 m^2^ g^−1^, and the micropore surface area accounts for 85% of the total area. The optimal conditions for achieving the maximum Cr(vi) adsorption capacity of 163.7 mg g^−1^ by the ACs were activation with a KOH/carbon ratio of 3.0, an initial Cr(vi) concentration of 400 mg L^−1^, an adsorbent dose of 2.0 g L^−1^ and pH of 4.5. Therefore, the ACs exhibit excellent adsorption performance for removing Cr(vi) from aqueous solutions. According to an investigation of the adsorption process, the adsorption isotherm is most consistent with the Langmuir isotherm model, and the adsorption kinetics were well described by the pseudo-second-order model.

## Abstract 2

A series of micro–mesoporous activated carbons (ACs) were prepared from sugar beet residue by a two-step method including KOH chemical activation and were used for Cr(vi) removal from aqueous solutions.

## Graphical abstract

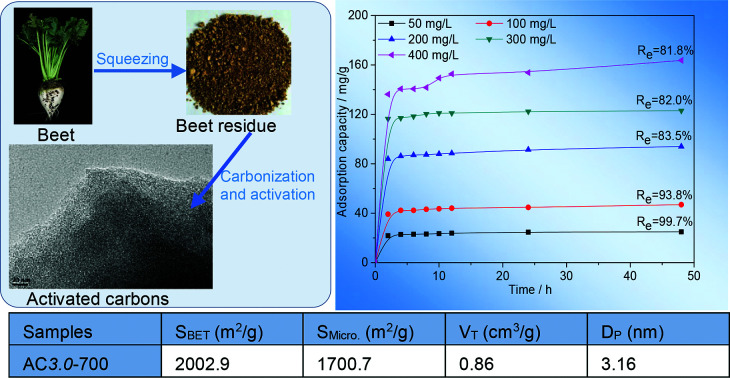

## Introduction

Chromium is a common metal pollutant mainly originating from industrial wastewater from electroplating, metallurgy, tanning, printing and dyeing processes.^1^ In general, the stable oxidation states of chromium are mainly Cr(vi) and Cr(iii).^2^ Cr(iii) is an essential trace element for humans. However, Cr(vi) is highly toxic and can lead to serious illness, dermatitis, and kidney and liver cancer, for example.^3^ The discharge limits of Cr(vi) for drinking water and inland surface water are 0.05 mg L^−1^ and 0.1 mg L^−1^, respectively.^4^ Hence, the removal of Cr(vi) from contaminated water is important for protecting the environment and human health.

The various conventional technologies for removing Cr(vi) from contaminated water include membrane processes,^5^ ion exchange,^6^ extraction,^7^ chemical precipitation,^8^ coagulation reduction,^9^ and adsorption.^10,11^ However, the disadvantages of most of these technologies are the high cost of equipment, massive consumption of energy and use of expensive chemicals. In addition, these traditional technologies are not suitable for removing toxic metals, which may cause secondary pollution. Among these methods, the activated carbon (AC) adsorption technique is the most extensively utilized method due to its excellent adsorption capacity, extended surface area, low cost, relatively easy regeneration and excellent physical and chemical properties.

Currently, agricultural wastes and biomass, such as shaddock peels,^12^ chestnut oak shells,^13^ coconut shell,^14^ corncob,^15^ sunflower seed husk,^16^ and peanut shell,^17^ are very important and economic raw materials for preparing AC. Sugar beet planting and the sugar industry play a very important role in northeast China. Beet residue, which is the by-product of sugar production, is mostly composed of cellulose, pentosan and lignin.^18,19^ In the countryside of Heilongjiang Province, China, a large amount of beet residue with low commercial value is produced every year and is mostly used as poultry feed, resulting in waste. The transformation of beet residue into products with economic value has good prospects because it utilizes agricultural waste as a resource. It is feasible to prepare biomass-based AC from beet residue as an adsorbent. Some researchers prepared activated carbon using beet residue as the raw material with different activators, such as phosphoric acid,^20,21^ concentrated sulfuric acid,^22^ and ZnCl_2_,^23,24^ and investigated its adsorption performance. However, no studies of the synthesis of AC from beet residue using KOH as the activator have been reported.

In this paper, a series of ACs were prepared using beet residue as the starting material and KOH as the activator. The resulting material has a high specific surface area and abundant pore structure. The effects of the activation temperature and the ratio of KOH to beet residue on the properties of the AC were studied. The ACs were used to remove Cr(vi) from aqueous solutions, and the adsorption kinetics and isotherms were also studied.

## Materials and methods

### Materials

Beet residue was obtained from the sugar refinery in Heilongjiang, China. The beet residue was crushed and dried at 110 °C for 48 h. The treated material was sieved, and particles in the size range of 0.3–0.8 mm were selected for the experiments.

### Preparation of the ACs

The ACs were prepared by a two-step procedure. First, the treated beet residue was placed in a ceramic crucible and carbonized in an N_2_-filled muffle tube furnace at 600 °C for 1 h after heating at a rate of 5 °C min^−1^. Second, the carbonized materials were chemically activated by mixing with KOH (AR, Sigma-Aldrich) in KOH/carbonized material ratios of 1 : 1, 2 : 1, 3 : 1 and 4 : 1 and then calcining at 700 °C under nitrogen gas at a 100 mL min^−1^ flow rate for 1.5 h. The prepared ACs were washed with a 0.1 M HCl solution until the pH reached 7. The ACs were dried at 100 °C overnight. The ACs were denoted AC-*x*-700, where *x* is the KOH/carbonized material ratio.

To determine the effect of the calcination temperature on the adsorption properties of the ACs, a series of ACs were prepared at different calcination temperatures. These ACs were denoted AC-3.0-*y*, where *y* is the calcination temperature.

### Characterization

The surface morphologies and microstructures of the ACs were observed by scanning electron microscopy (SEM) and transmission electron microscopy (TEM) with Leo 1430 and Tecnai G220 S-Twin instruments. The crystal structures of the ACs were determined by a powder X-ray diffractometer (XRD, D8 ADVANCE of Bruker). The specific surface areas and pore volumes of the ACs were measured at −196 °C using a Quantachrome AUTOSORB-1-MP porous materials analyser. The specific surface area (*S*_BET_) was calculated by the Brunauer–Emmett–Teller (BET) method. The *t*-plot method was employed to calculate the micropore volumes (*V*_*t*-plot_) and micropore surface areas (*S*_micro_). The Barrett–Joyner–Halenda (BJH) method was used to evaluate the mesopore volume (*V*_BJH_). The functional groups on the surfaces of the beet residue and ACs was analysed by a FTIR spectrometer (Vertex 70, Bruker). The Raman spectra were recorded by a Jobin Yvon HR 800 micro-Raman spectrometer with a 458 nm laser in the wavelength range of 1000 to 2000 cm^−1^.

### Adsorption experiments

To prepare Cr(vi) solutions with different predetermined concentrations, K_2_Cr_2_O_7_ (AR, Sigma-Aldrich) was dissolved in deionized water. 0.1 g ACs were mixed with a 50 mL Cr(vi) solution with different concentration (50, 100, 200, 300 and 400 mg L^−1^). The initial pH of the Cr(vi) solution was regulated to 4.5 using HCl and NaOH (1 mol L^−1^) solutions, and then the mixture was constantly stirred at 25 °C. The supernatant was obtained by filtration at preset time intervals until adsorption equilibrium was achieved, and the Cr(vi) content of the samples was measured.

The residual Cr(vi) concentrations were measured by a UV/VIS spectrophotometer (PE, Lambda12) at a wavelength of 540 nm.

The removal efficiency (*R*_e_, %), adsorption capacity (*q*_*t*_, mg g^−1^) and equilibrium adsorption capacity (*q*_e_, mg g^−1^) were calculated as follows:1
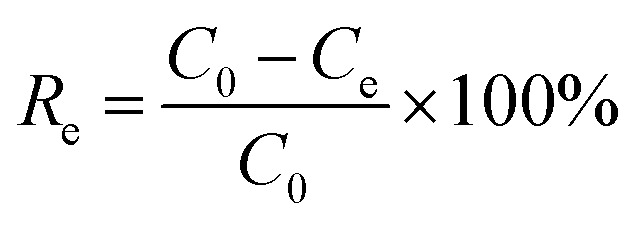
2
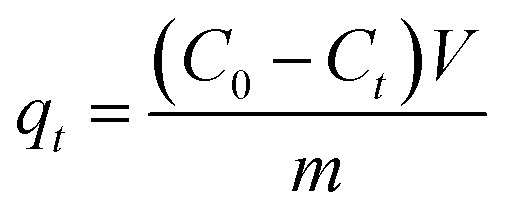
3
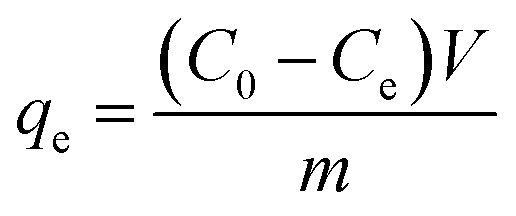
*C*_0_ (mg L^−1^), *C*_e_ (mg L^−1^) and *C*_*t*_ (mg L^−1^) are the concentrations of Cr(vi) initially, at equilibrium and at time *t*, respectively. *V* (L) is the Cr(vi) solution volume, and *m* (g) is the mass of the adsorbent used.

### Adsorption isotherms and kinetic models

An adsorption isotherm is used to represent the distribution of Cr(vi) in the liquid phase and solid phases in the equilibrium state.^17^ Thus, three common isotherm models, namely, the Freundlich (eqn (4)), Langmuir (eqn (5)) and Temkin (eqn (6) and (7)) models were used to analyse the adsorption equilibrium of Cr(vi) on the ACs. The equilibrium parameters were derived from adsorption experiments using AC-3.0-700 as the adsorbent with various Cr(vi) concentrations at 25 °C, an adsorbent dose of 2.0 g L^−1^ and pH of 4.5.

The equations are given as follows:

4 *q*_e_ = *K*_F_*C*_e_^1/*n*^

5
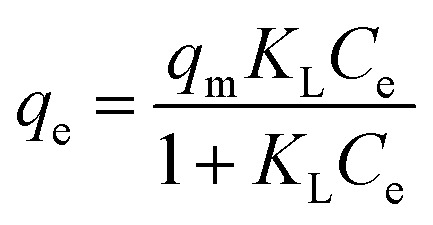

6 *q*_e_ = *B* ln(*K*_T_*C*_e_)

7
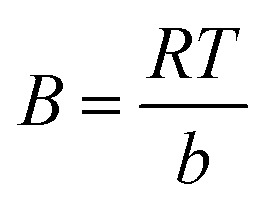
Here, *q*_m_ (mg g^−1^) is the maximum adsorption capacity, and *K*_F_ (L mg^−1^), *K*_L_ and *K*_T_ are the Freundlich, Langmuir and Temkin constants, respectively. *B* is the heat of adsorption constant in the Temkin model. Gas constant (*R*) is 8.314 J mol^−1^ K^−1^ and *T* (K) is the absolute temperature.^25–27^

The dynamics of the Cr(vi) adsorption process were investigated using three kinetic models, *i.e.*, the pseudo-first-order model (eqn (8)), pseudo-second-order model (eqn (9)) and Weber–Morris intraparticle diffusion (eqn (10)).

8 ln(*q*_e_ − *q*)*t* = ln *q*_e_ − *k*_1_*t*

9
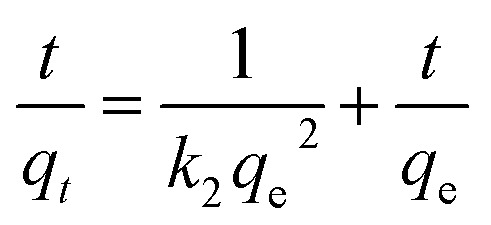

10 *q*_*t*_ = *k*_id_*t*^1/2^ + *C*

Here, *k*_1_ (h^−1^) and *k*_2_ (g mg^−1^ h^−1^) are the pseudo-first- and pseudo-second-order model rate constants, respectively; *k*_id_ (mg g^−1^ min^−1/2^) is the intraparticle diffusion rate constant; and *C* (mg g^−1^) is the model constant for the boundary layer thickness.^28–30^

## Results and discussion

### Characterization of the ACs

#### Morphology of the ACs

**Fig. 1:**
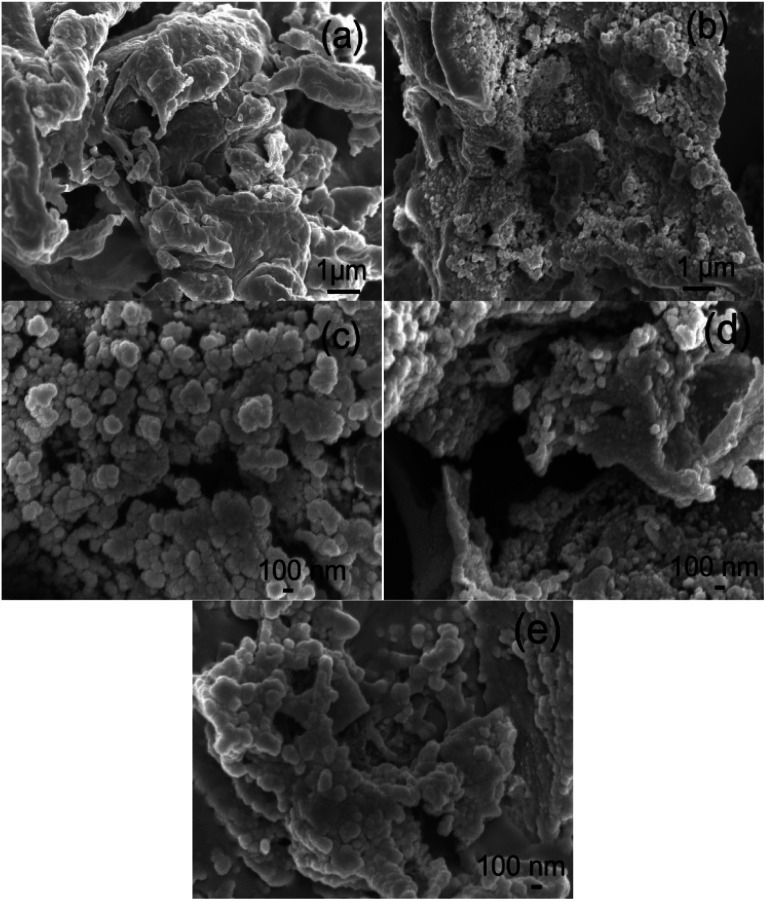
SEM images of the beet residue (a), AC-1.0-700 (b), AC-2.0-700 (c), AC-3.0-700 (d) and AC-4.0-700 (e).

**Fig. 2:**
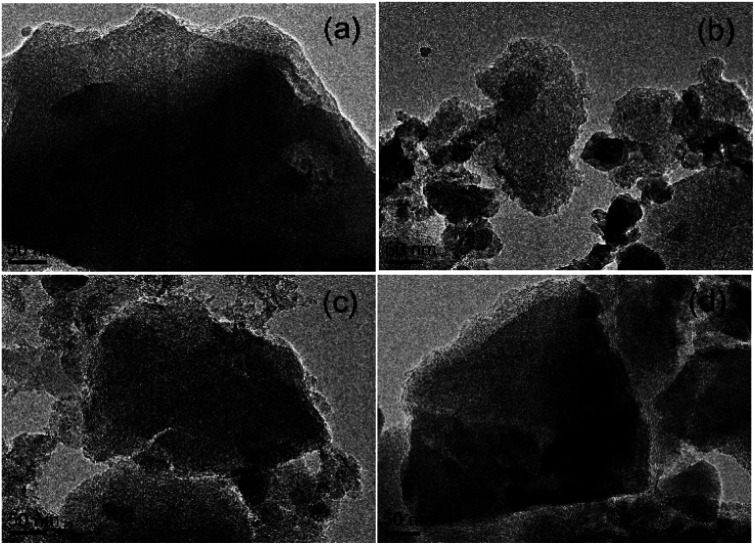
TEM images of (a) AC-1.0-700, (b) AC-2.0-700, (c) AC-3.0-700 and (d) AC-4.0-700.

SEM and TEM images of the morphologies of the beet residue and ACs are shown in Fig. 1 and 2. The beet residue has an irregular bulk shape and relatively smooth surface without an obvious pore structure, whereas the activated and carbonized AC samples are obviously dehydrated and granulated and have an abundant pore structure derived from the accumulation of flake-like carbon. The SEM image shows that the size of a single particle is about 150–200 nm. The TEM images further confirm that the AC samples have an excellent pore structure, and they clearly show the abundant micropores in the ACs.

### N_2_ adsorption–desorption analysis

**Fig. 3:**
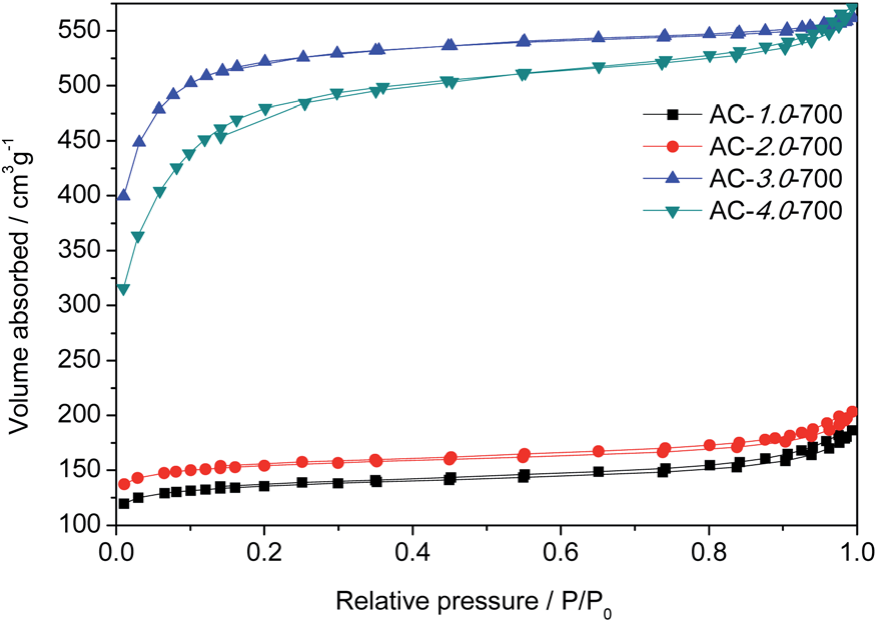
N_2_ adsorption and desorption isotherms of the AC samples.

The specific surface areas and pore structures of the ACs were generally measured by N_2_ adsorption–desorption, and the spectra and corresponding textural parameters are shown in Fig. 3 and Table 1. According to the IUPAC classification, the measured ACs exhibited a type I nitrogen adsorption–desorption isotherm due to its rich microporous structure.^3^Table 1 gives the details of the textural characteristics of the ACs. The BET surface area increased gradually with increasing KOH amount. The highest BET surface area and the total pore volume (*V*_T_) of the beet residue-derived carbons reached 2002.9 m^2^ g^−1^ and 0.86 cm^3^ g^−1^. For the AC-3.0-700 sample, the microporous specific surface accounts for 85% of the total specific surface, confirming the well-developed microporous structure,^31^ which could provide abundant adsorption sites.^32^

**Table 1:** The porous structure properties of AC samples

Samples	*S*_BET_ (m^2^ g^−1^)	*S*_micro_ (m^2^ g^−1^)	*V*_T_ (cm^3^ g^−1^)	*V*_*t*-plot_ (cm^3^ g^−1^)	*V*_BJH_ (cm^3^ g^−1^)	*D*_P_ (nm)
AC-1.0-700	515.4	417.7	0.28	0.17	0.10	7.07
AC-2.0-700	588.4	488.7	0.30	0.20	0.10	6.91
AC-3.0-700	2002.9	1700.7	0.86	0.67	0.13	3.16
AC-4.0-700	1774.6	1077.9	0.87	0.43	0.30	3.33

### XRD

**Fig. 4:**
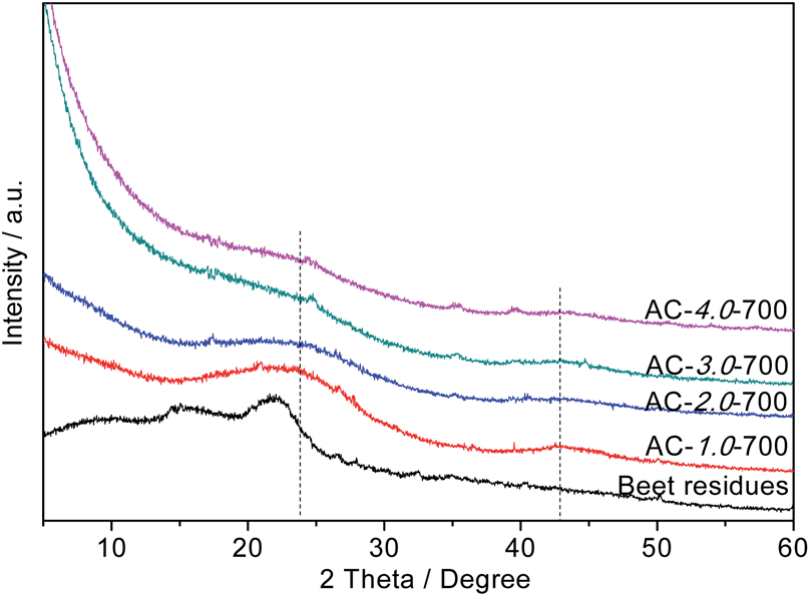
XRD patterns of the beet residue and AC samples.

The crystallization degrees of the beet residue and ACs were evaluated by XRD analysis. As shown in Fig. 4, the two typical diffraction peaks of the ACs appear at 2*θ* = 24° and 2*θ* = 42° and correspond to the (002) and (100) surface planes, respectively. These strong diffraction peaks indicate that the ACs contain many graphitic crystals, whereas a weak peak would indicate that the ACs only exhibit partial graphitization characteristics and contain very few graphitic crystals.^33^ As the KOH ratio of the AC samples increases, the peak intensity decreases, and the graphitization degree also decreases. This phenomenon is consistent with the Raman spectral analysis.

### FT-IR spectroscopy

**Fig. 5:**
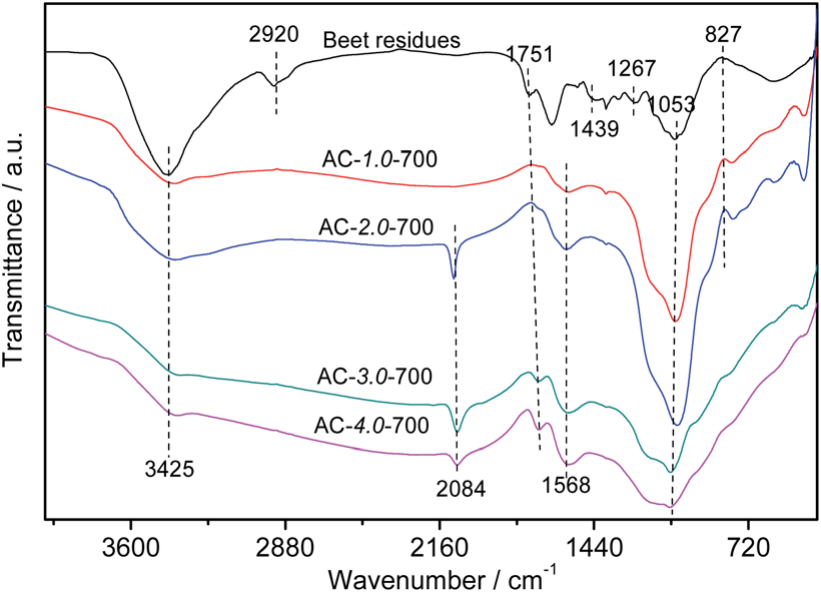
FTIR spectra of the beet residue and AC samples.

Fig. 5 shows the FTIR spectra of the beet residue and AC samples. The peak at 3425 cm^−1^ is related to the stretching vibration of –OH due to the lignin, cellulose and hemicellulose in the beet residue, which contain carboxyls, phenols and alcohols,^34^ and it decreases gradually with increasing amount of KOH. This trend showed that –OH on the surface of the beet residue was reduced after carbonization. The beet residue exhibits a distinct absorption peak at 2920 cm^−1^.^35^ The absorption peak represented the telescopic vibration of C–H. However, the peak intensity of the ACs gradually decreases, and the peak almost disappears. The results showed that cellulose was gradually decomposed after carbonization, which promotes the formation of the pore structure. The observed peaks at 1751 cm^−1^ are attributed to the C=O band of carbonyl groups.^36^ The peak intensity of the ACs is weaker than that of the beet residue, due to the degradation of hemicellulose during carbonization. The band at 1568 cm^−1^ represents the C=C stretching vibration of lignin. The band at 1439 cm^−1^ can be attributed to C–H bending vibrations in CH_2_ or CH_3_ groups.^37^ The band at approximately 1267 cm^−1^ is the C–O stretching in carboxylic groups or the C–O–C stretching vibration.^38^ The absorption peak of the –C=O stretching vibration in cellulose and hemicellulose appears at 1053 cm^−1^.^39^ The absorption peak strength of the ACs decreased, revealing that cellulose and hemicellulose could be sufficiently decomposed with increasing KOH and carbonization.

### Raman spectroscopy

**Fig. 6:**
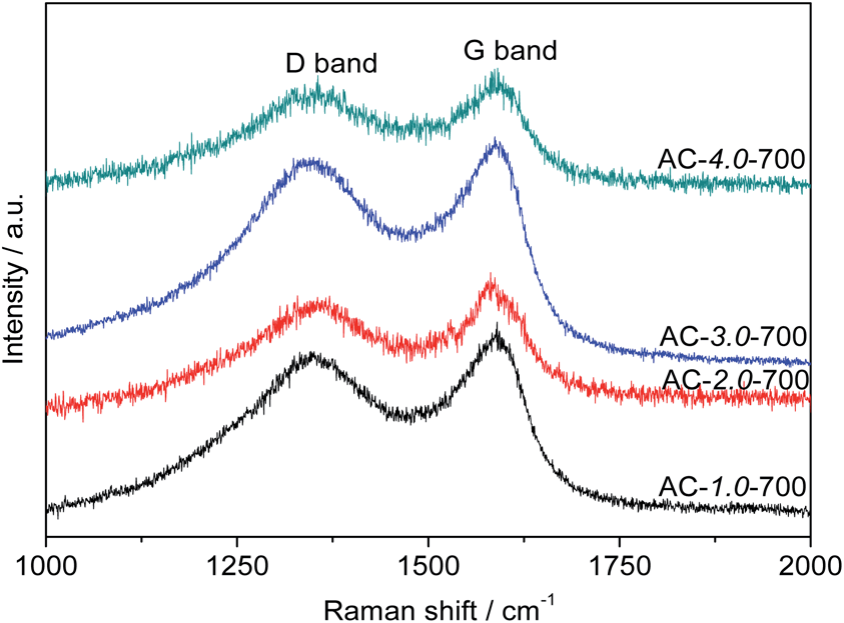
Raman spectra of the AC samples.

Fig. 6 presents the Raman spectra of the ACs. All the samples exhibited two obvious intrinsic peaks at approximately 1580 cm^−1^ and 1350 cm^−1^, corresponding to the G and D bands, respectively. The intensity ratio between the D and G bands (*I*_D_/*I*_G_) is generally used to indicate the degree of graphitization of carbon. The *I*_D_/*I*_G_ ratio is directly proportional to the degree of disorder in the carbon structure. The lower the *I*_D_/*I*_G_ ratio is, the higher the degree of graphitization is.^40^ The graphitization degree decreased gradually with increasing KOH amount. The *I*_D_/*I*_G_ ratios of AC-1.0-700, AC-2.0-700, AC-3.0-700 and AC-4.0-700 are 0.89, 0.91, 0.95 and 0.97, respectively. These results can be attributed to the etching effect of activation on the structure, resulting in structural defects and increased carbon disorder.

### Adsorption performance

#### Effect of the KOH/carbon ratio

**Fig. 7:**
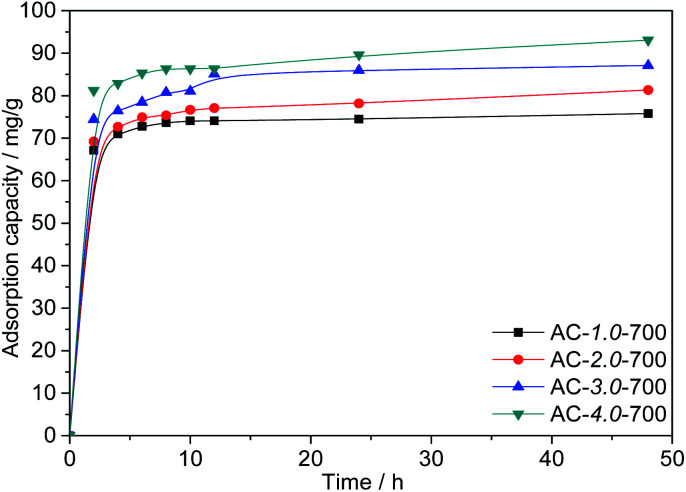
Adsorption capacities of the ACs with various KOH/carbon ratios.

Fig. 7 shows the adsorption capacities of AC samples prepared with different KOH/carbon ratios. As shown in the figure, as the KOH/carbon ratio increases from 1.0 to 4.0 at an the *C*_0_ value of 200 mg L^−1^, the Cr(vi) adsorption capacities of the ACs increase significantly from 75.79 mg L^−1^ to 93.07 mg L^−1^, which is directly related to the BET specific surface area. It is reasonable that the large specific surface area improves the adsorption performance. However, the high proportion of activating agent leads to a reduced yield of activated carbon. Therefore, AC-3.0-700 was chosen for the following experiment.

#### Effect of the activation temperature

**Fig. 8:**
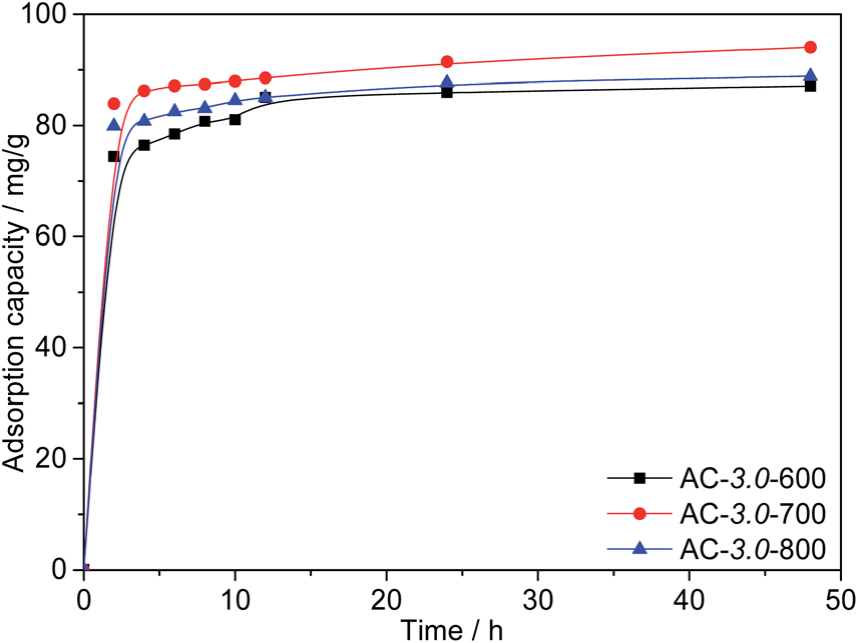
Adsorption capacities of ACs with various activation temperatures.

Fig. 8 shows the Cr(vi) adsorption capacities of the ACs at different activation temperatures. The results show that high temperatures are conducive to the diffusion of KOH into the precursor and the subsequent formation of abundant porosity. This is consistent with the research results of other scholars.^41^ However, the adsorption capacity of AC-3.0-800 decreased when the activation temperature increased to 800 °C. The excessive activation temperature causes the expansion of the micropores and subsequent formation of mesopores and macropores,^42^ resulting in a slight decrease in the BET surface area. The maximum adsorption capacity of the ACs for Cr(vi) reached 94.0 mg g^−1^ at a KOH/carbon ratio of 3.0, activation temperature of 700 °C and the *C*_0_ value of 200 mg L^−1^.

#### Effect of initial Cr(vi) concentration

**Fig. 9:**
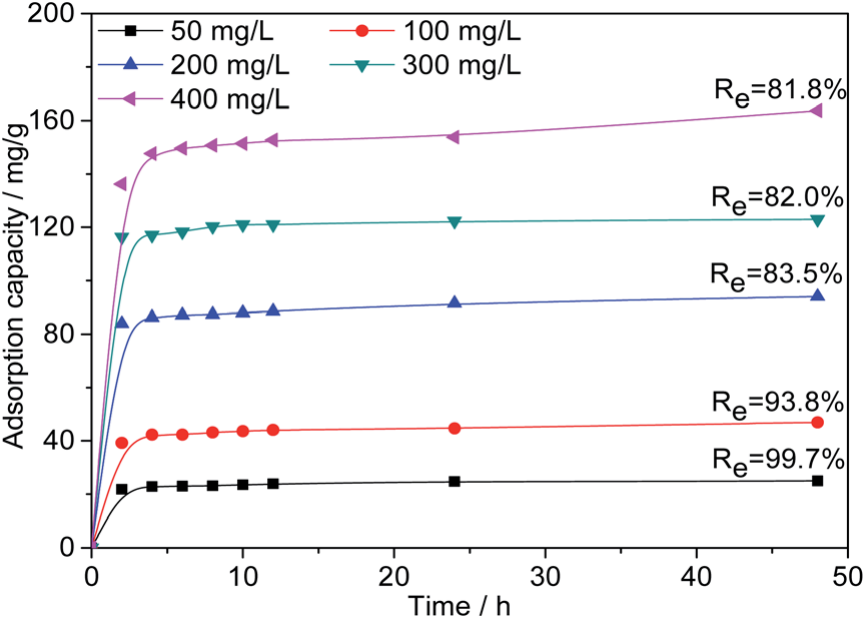
Effects of contact time and initial concentration on the adsorption capacity of Cr(vi) on AC-3.0-700.

As shown in Fig. 9, to further investigate the adsorption capacity of AC-3.0-700, the Cr(vi) adsorption of AC-3.0-700 was measured at varying initial concentrations. The Cr(vi) adsorption capacity of AC-3.0-700 increases rapidly in the beginning (0–2 h) and then increases gradually with time until adsorption equilibrium is achieved.

The existence of many available active adsorption sites on the activated carbon is the reason for the high adsorption rate at the beginning of adsorption. Over time, the available active adsorption sites were gradually occupied, which leads to a decrease in the adsorption rate. As shown in Fig. 9, increasing the *C*_0_ value from 50 mg L^−1^ to 400 mg L^−1^ led to a linear increase in the Cr(vi) adsorption capacity of the prepared AC-3.0-700 from 24.9 mg L^−1^ to 163.7 mg L^−1^, indicating that the surface of the activated carbon had abundant micropores and mesopores due to the KOH activation and carbonization treatment, and it exhibited an excellent adsorption performance. This adsorption capacity of the ACs is generally higher than the results reported in the current literature, such as corn stalks-derived AC adsorption capacity of 89.8 mg g^−1^,^43^ olive bagasse ACs of 126.67 mg g^−1^,^44^ longan seed ACs of 35.02 mg g^−1^,^45^ bagasse ACs of 80.8 mg g^−1^^46^ and eucalyptus sawdust ACs of 45.88 mg g^−1^,^47^*etc.* In addition, the removal rate (*R*_e_) of Cr(vi) decreased from 99.7% to 81.8%, because as the *C*_0_ value increases, the Cr(vi)/AC ratio increased, leading to the competition for active sites and the attainment of the saturation state.^48^

### Kinetic studies

**Fig. 10:**
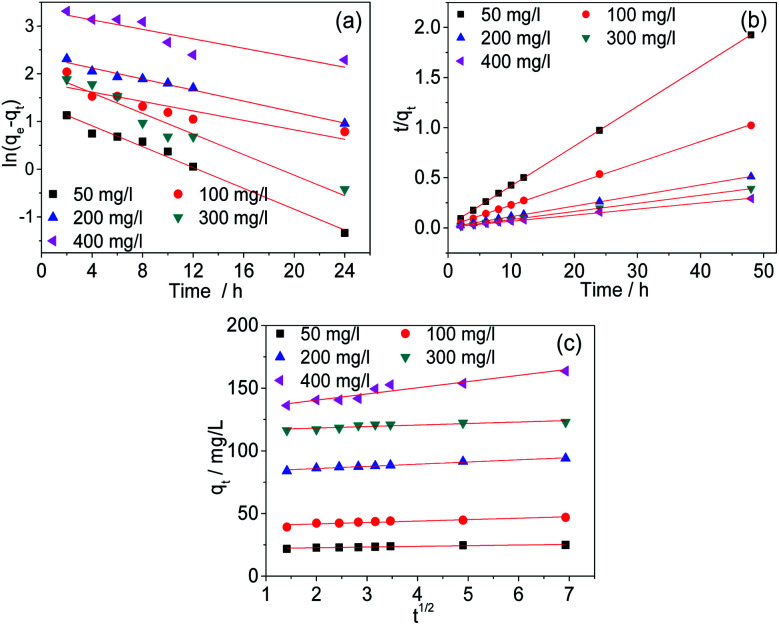
Kinetic curves of pseudo-first order (a), pseudo-second order (b) and Weber–Morris models (c) of AC-3.0-700.

The kinetics were studied to investigate the physical and chemical characteristics of the adsorption processes. In this study, the experimental data were analysed with the pseudo-first-order, pseudo-second-order and Weber–Morris models. The fitted curves are shown in Fig. 10. The fitting parameters were listed in Table 2. The results indicate that the adsorption process of Cr(vi) by AC-3.0-700 can be described properly by the pseudo-second-order model, because the *R*^2^ value is the highest and the calculated adsorption capacity (*q*_e,cal_) is comparatively similar to the practical experimental adsorption capacity (*q*_e,exp_). Therefore, the adsorption of Cr(vi) by the AC-3.0-700 adsorbent is predominantly controlled by chemisorption.^3^

**Table 2:** Adsorption kinetic parameters for Cr(vi) removal by AC-3.0-700

Kinetics/concentration	Concentration (mg L^−1^)				
	50	100	200	300	400
*q*_e,exp_	24.93	46.91	94.03	122.94	163.68

**Pseudo-first order**					
*q*_e,cal_	3.84	6.14	10.50	7.62	27.86
*k*_1_	0.10918	0.04951	0.05776	0.10774	0.04958
*R* ^2^	0.98283	0.76039	0.97992	0.93996	0.74521

**Pseudo-second order**					
*q*_e,cal_	25.19	47.26	94.79	123.46	165.56
*k*_2_	0.07210	0.02919	0.01795	0.03882	0.00602
*R* ^2^	0.99989	0.99946	0.99972	0.99999	0.99897

**Weber–Morris**					
*C*	21.63523	39.35885	82.41067	115.79862	130.59249
*k*_id_	0.5386	1.15705	1.7455	1.2081	4.94184

### Adsorption isotherm

**Fig. 11:**
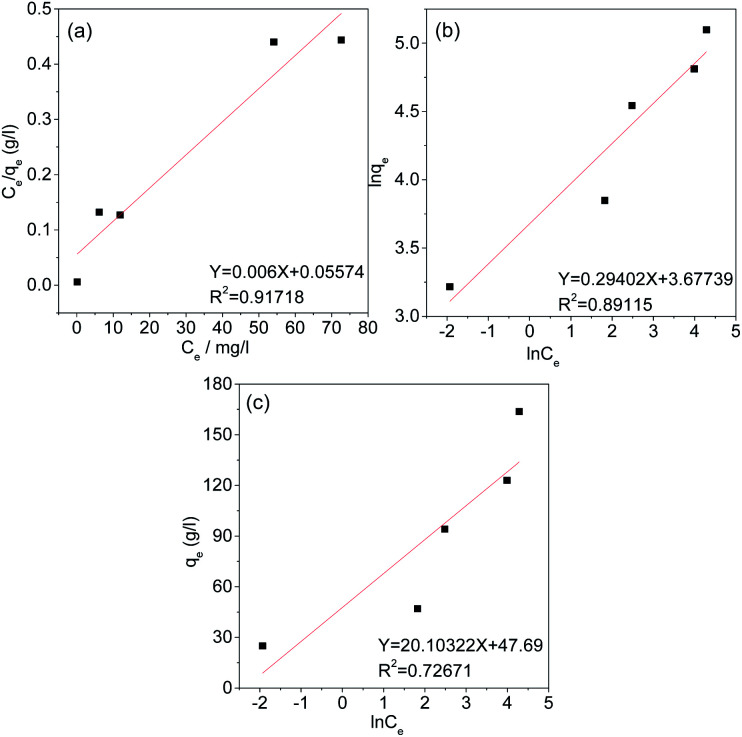
Langmuir (a), Freundlich (b), and Temkin (c) Isotherms of AC-3.0-700.

Under equilibrium conditions, adsorption isotherms can be used to better understand the distribution of the adsorbate between the aqueous and solid phases.^49^ Therefore, the type of adsorption of Cr(vi) on the AC was studied using the Langmuir, Freundlich and Temkin isotherm models. The fitting curves and related parameters are shown in Fig. 11 and Table 3. The results indicate that the adsorption isotherm is most consistent with the Langmuir isotherm model, and the maximum adsorption capacity (166.667 mg g^−1^) calculated by the Langmuir isotherm model is similar to the experimental maximum adsorption capacity (163.7 mg g^−1^). This result implies that the adsorption of Cr(vi) by AC-3.0-700 from aqueous solutions is monolayer adsorption, Cr(vi) has a uniform distribution, and no transmigration occurs on the activated carbon surface.^50^

**Table 3:** Isotherm parameters of AC-3.0-700

Isotherm model	Parameter	
Langmuir model	*R* ^2^	0.91718
	*K*_L_ (L mg^−1^)	0.01076
	*q*_m_ (mg g^−1^)	166.667
Freundlich model	*R* ^2^	0.89115
	*K*_F_ (mg g^−1^(L mg^−1^)^−1/*n*^)	39.54
	1/*n*	0.29402
Temkin model	*R* ^2^	0.72671
	*K*_T_ (L g^−1^)	10.721
	*B*	20.10322

## Conclusions

Activated carbon with a large BET surface area and abundant pore structure was prepared by a two-step KOH activation method, and the agricultural waste beet residue was used as the raw material. The characterization tests indicate that well-developed micropores can be formed, and the BET surface area and total pore volume of the beet residue-derived AC reached maxima of 2002.9 m^2^ g^−1^ and 0.86 cm^3^ g^−1^ at a beet residue/KOH ratio of 3.0 and calcination temperature of 700 °C. The beet residue-derived ACs exhibited a remarkable removal ability for Cr(vi) with a maximum adsorption capacity of 163.7 mg g^−1^ at 25 °C. The adsorption isotherms are most consistent with the Langmuir isotherm model, and the adsorption kinetics were well represented by the pseudo-second-order model. The results indicate that the adsorption process of Cr(vi) was based on monolayer physical adsorption. In conclusion, beet residue could be considered to be an efficient biomass material for preparing activated carbon with excellent applications for the treatment of wastewater.

## Conflicts of interest

There are no conflicts to declare.

## Supplementary Material
